# Cerebral DWI lesion burden following carotid artery stenting with guided antiplatelet therapy: a retrospective comparative study

**DOI:** 10.1186/s42155-026-00711-w

**Published:** 2026-05-29

**Authors:** Pavol Vigláš, Jan Raupach, Aleš Hejčl, David Černík, Karel Hrach, Patrik Matras, Pavla Bradáčová, Pavel Ryška, Filip Cihlář

**Affiliations:** 1 Department of Radiology, Faculty of Medicine in Hradec Králové, Charles University, Šimkova 870, Hradec Králové, 500 05 Czech Republic; 2 Department of Radiology, J. E. Purkyně University - Faculty of Health Studies and Krajská zdravotní – Masaryk Hospital, Sociální péče 3316/12A, Ústí Nad Labem, 401 13 Czech Republic; 3 Department of Radiology, University Hospital Hradec Kralove, Sokolská 581, Hradec Králové, 500 05 Czech Republic; 4 Department of Neurosurgery, J. E. Purkyně University - Faculty of Health Studies and Krajská zdravotní – Masaryk Hospital, Sociální péče 3316/12A, Ústí Nad Labem, 401 13 Czech Republic; 5 Department of Neurology, Krajská zdravotní – Masaryk Hospital, Sociální péče 3316/12A, Ústí Nad Labem, 401 13 Czech Republic; 6 Department of Neurology, Palacký University Medical School and University Hospital, Zdravotníků 248/7, Olomouc, 779 00 Czech Republic; 7 Department of Biomedicine and Laboratory Diagnostics, J. E. Purkyně University– Faculty of Health Studies and Krajská zdravotní – Masaryk Hospital, Sociální péče 3316/12A, Ústí Nad Labem, 401 13 Czech Republic; 8 Department of Clinical Heamatology, Krajská zdravotní – Masaryk Hospital, Sociální péče 3316/12A, Ústí Nad Labem, 401 13 Czech Republic

**Keywords:** Carotid stenting, Antiplatelet therapy, New ischemic lesion, Silent ischemia, Diffusion-weighted image

## Abstract

**Purpose:**

To analyze the number, location, and clinical significance of new ischemic lesions (NIL) after carotid stenting in patients with guided antiplatelet therapy. To evaluate factors leading to thromboembolic complications and silent ischemia. Secondarily, we compared the incidence of NIL among historical cohorts with guided and standard antiplatelet therapy.

**Methods:**

We conducted a retrospective analysis of prospectively collected data from 171 patients who underwent carotid stenting in a single center between 2014 and 2023. Magnetic resonance imaging with diffusion-weighted images was performed before and after stenting. We evaluated NIL after the procedure and their dependence on patient demographics, degree of stenosis, instrumentation used, and antiplatelet therapy. The incidence of NIL was compared to three cohorts receiving more potent or guided antiplatelet therapy based on platelet function test (*n* = 689) and to seven cohorts receiving standard dual antiplatelet therapy with aspirin and clopidogrel (*n* = 2777), using the meta-analytical approach.

**Results:**

New ischemic lesions were found in 15.8% of patients (27/171). We recorded five ischemic strokes (2.9%). Most NIL were located in the ipsilateral middle cerebral artery basin (91%), but they also occurred contralaterally or in another basin. Stenosis length ≥ 15 mm (*p* = 0.012) was shown to be significantly associated with the development of NIL. The incidence of NIL in a center with guided antiplatelet therapy was distinctly lower compared to results of the studies with standard antiplatelet therapy, 15.8% vs. 28.3–51.3%.

**Conclusion:**

Guided antiplatelet therapy in carotid stenting is safe and shows a lower incidence of new ischemic lesions on MR-DWI compared to results with standard antiplatelet therapy. In this study with guided antiplatelet therapy, NIL were only associated with greater stenosis length.

## Introduction

Worldwide, stroke is identified as the second main cause of death and the primary reason for disability, while atherosclerotic stenosis of the internal carotid artery causes approximately 20% of ischemic strokes [1, 2].

Carotid artery stenting (CAS) is currently a proven method of treatment for carotid stenosis. CAS is a safe alternative to carotid endarterectomy (CEA) with comparable long-term results [3–9]. However, the spectrum of periprocedural complications differs between CAS and CEA. While thromboembolic complications are more common after CAS, myocardial infarction and cranial nerve paresis are reported to occur more frequently after CEA [6–9]. New ischemic lesions (NIL) visualized by diffusion-weighted imaging are more common after CAS compared to CEA [8, 9].

In addition to acute NIL that cause stroke or transitory ischemic attack, new ischemic lesions may be asymptomatic, i.e., silent. It has been shown that even silent ischemia caused by carotid artery stenting can cause dementia, cognitive decline, and a higher risk of stroke in the future [10, 11]. The reduction of periprocedural thromboembolic complications is considered another milestone in carotid stenting [10–12]. While guided antiplatelet therapy has proven safe and beneficial in reducing thromboembolic complications in stent-assisted intracranial coiling, its role has not yet been established in carotid stenting [13–17]. Several studies suggest that routine testing of the efficacy of antiplatelet therapy prior to CAS and changing treatment in cases of in-vitro resistance reduces the incidence of thromboembolic complications [15–17]. However, the current ESVS guidelines did not find sufficient evidence to support the routine use of platelet function testing in this context, and its role in carotid stenting remains to be established in prospective studies [18].

## Material and methods

From January 2014 to December 2023, a total of 286 patients underwent elective extracranial carotid stenting at the Radiology department of Masaryk Hospital Ústí nad Labem for stenosis caused by atherosclerosis or for restenosis after previous carotid endarterectomy. Patients treated with CAS for other reasons (acute stenting for tandem occlusion, carotid dissection) were not included in this cohort. Of the total number, 171 patients underwent brain imaging using DWI before and after carotid intervention. These patients were included in the retrospective analysis.

We primarily analyzed the number, size, localization, and clinical significance of new ischemic lesions (NIL) and evaluate risk factors related to demographic, lesion and procedural characteristics.

Secondarily, the incidence of NIL was compared to the incidence in the largest historical cohorts. These comparative studies were searched in the MEDLINE (PubMed) and SCOPUS databases. A combination of the following keywords was used: “carotid stenosis,” “carotid artery stenting,” “CAS,” “carotid angioplasty,” “ischemic lesion,” “cerebral embolism,” “diffusion-weighted imaging,” “DWI,” “magnetic resonance imaging,” and “MRI.” Studies were eligible for inclusion if they reported the incidence of new ischemic lesions on DWI after carotid artery stenting and provided traceable data on antiplatelet therapy regimen and procedural characteristics. The ten largest studies fulfilling these criteria were selected for comparison. Among them are five prospective monocentric studies, two are retrospective monocentric studies, one prospective multicenter study, and one retrospective multicenter study and one meta-analysis that examined the incidence of NIL in 20 studies was also included.

### Study criteria

Pre-procedural computed tomography angiography was routinely performed in all patients to evaluate the degree and morphology of carotid stenosis, assess vascular and aortic arch anatomy, and plan the stenting procedure. CAS was performed in symptomatic patients if the degree of stenosis was more than 50%, and in asymptomatic patients if it was more than 70%. The degree of stenosis was determined according to North American Symptomatic Carotid Endarterectomy Trial criteria [1]. Informed consent was obtained. Assessment of clinical symptomatology was performed by the referring neurologist or neurosurgeon. We considered symptomatic patients as those with evidence of cerebral ischemia, TIA or amaurosis fugax in the previous 6 months. Inclusion criteria included available pre- and post-procedural MR-DWI and platelet function test results.

Contraindications to stenting in the indication analysis were marked tortuosity of the arteries, inability to achieve safe vascular access, and a circularly calcified lesion. Patients treated with CAS for other reasons (tandem occlusion, carotid dissection) were not included in this study.

### Antiplatelet therapy regimen

Dual antiplatelet therapy (DAPT) in our center was commenced at least 5 days prior to the procedure, utilizing daily doses of 100 mg acetylsalicylic acid and 75 mg clopidogrel. All patients were tested for antiplatelet therapy efficacy as part of their preoperative examination or upon admission to the hospital. Platelet function was analyzed using certified laboratory techniques, including light transmission aggregometry (LTA) (*n* = 109) and the Multiplate® analyzer (*n* = 62). If resistance to clopidogrel was detected, treatment was changed to ticlopidine, which was administered at a dose of 2 × 250 mg daily for at least 5 days prior to the procedure. After its withdrawal from the market in 2020, it was replaced by ticagrelor. For ticagrelor, a loading dose of 180 mg (2 × 90 mg) was administered. The efficacy of the changed treatment was also tested. If resistance to clopidogrel and ticagrelor (or ticlopidine) was detected, a loading dose of 30 mg of prasugrel was administered. After carotid stent implantation, DAPT is recommended for at least 3 months at maintenance doses of 100 mg ASA and either 75 mg clopidogrel (daily) or 90 mg ticagrelor (twice daily), or 5 mg prasugrel (daily). The efficacy of acetylsalicylic acid was also routinely tested to complete the overall picture of platelet function. ASA is currently used as a permanent component of DAPT. It has not yet been proven that higher doses of ASA reduce the incidence of thromboembolic events. For this reason, aspirin therapy was not modified within our treatment protocol, and our attention was focused on adjustment of P2Y12 inhibitor therapy [19]. Therefore, our attention has focused solely on alternatives to clopidogrel.

Light transmission aggregometry (LTA) was performed in platelet-rich plasma (PRP) via the turbidimetric method using an APACT 4004 aggregometer (LABiTec, Ahrensburg, Germany). Venous blood was collected into 0.109 M sodium citrate at a 1:9 ratio. To obtain PRP, samples were centrifuged at 150 g for 10 min; platelet-poor plasma (PPP), used as a reference blank, was prepared by centrifugation at 2500 g for 10 min. The target platelet concentration for PRP was established at 150–600 × 10⁹/L. Samples exceeding 600 × 10⁹/L were adjusted to 350 × 10⁹/L using physiological saline, while those below 150 × 10⁹/L were processed with a caveat regarding potential interference from low platelet density. LTA was induced using adenosine diphosphate (ADP, 4 µmol/L final concentration) and arachidonic acid (AA, 1 mmol/L final concentration; Hyphen BioMed, Paris, France). For each assay, 140 µL of PRP was added to a cuvette pre-tempered to 37 °C and stirred magnetically. Aggregation was initiated by the addition of 20 µL of agonist under constant stirring at 250 g. The resulting increase in light transmission, reflecting platelet aggregate formation, was monitored for 10 min and expressed as maximum aggregation (MoA, %). Therapeutic efficacy for acetylsalicylic acid was defined as MoA ≤ 20% (AA-induced), while high on-treatment platelet reactivity (HTPR) to P2Y₁₂ inhibitors was defined as MoA ≥ 60% (ADP-induced). Parallel impedance aggregometry was conducted using the Multiplate® analyzer (Roche Diagnostics, Mannheim, Germany). Whole blood was collected in hirudin-coated tubes (Monovette-S, Sarstedt, Nümbrecht, Germany) and allowed to stabilize at room temperature. For the ASPI and ADP tests, 300 µL of blood was diluted with pre-warmed (37 °C) isotonic saline and incubated for 3 min. Following the addition of 20 µL of the respective agonist, impedance changes were recorded for 6 min. Results were quantified as aggregation units (AU), aggregation velocity (AU/min), and area under the curve (AUC, U); the latter served as the primary analytical parameter. Reference thresholds for hirudin-anticoagulated samples were < 45 U for the ADP test and < 30 U for the ASPI test.

### Carotid stenting procedure

The CAS procedure was performed on the Philips Allura Xper FD20 angiography system by three operators with 10–20 years of experience in vascular interventional radiology. Complications were proportionally distributed among the operators. The operator with the greatest experience and the highest number of CAS procedures performed (*n* = 188) had a lower number of NIL events (*n* = 12) compared with operators who had performed fewer CAS procedures (*n* = 56 and *n* = 22), with 10 and 4 NIL events recorded, respectively. A chi-square test of independence showed a significant difference in NIL event rates among operators (*p* = 0.015), however, due to small subgroup sizes, results should be interpreted with caution.

Selective angiography of the common carotid artery was conducted in various projections to assess stenosis, with mandatory posteroanterior and lateral digital subtraction angiography views including the entire skull. The contrast medium was injected at a rate of 4 ml/s. Heparin was administered at 70 units/kg IV bolus, with a maximum of 5000 units, and protamine was not used after the procedure. A Filter Wire EZ served as a distal thromboembolic protection device. The treatment for stenosis was determined by the operator. If the internal carotid artery diameter at the stenosis was 1 mm or less, predilatation (with a balloon size of 2 or 3 mm) was performed to facilitate proper stent expansion. A self-expandable carotid stent, ranging from 6 to 10 mm in diameter and 20 mm to 40 mm in length, was then placed. Post-dilatation with a maximum balloon diameter of 5 mm was conducted, as larger balloons increase the risk of plaque protrusion without improving hemodynamics. Atropine (0.5 mg IV bolus) was selectively administered based on the patient’s response to predilatation, more specifically if bradycardia occurred with a heart rate below 40 beats per minute. The procedure was successful when a maximum of 30% residual stenosis was achieved. A brief neurological examination by the operator was always performed before the removal of the guiding sheath. Hemostasis at the femoral access site was achieved in most cases with the AngioSeal closure device (Terumo, USA).

### Imaging protocol

Magnetic resonance imaging in our center was performed 1 to 5 days before the procedure, and 1 to 3 days after the procedure. The MRI scans were evaluated by radiologists with more than 10 years of experience. MRI was performed on a 3 T Ingenia (Philips, Best, The Netherlands). New ischemic lesions were defined as hyperintense lesions on DWI sequences that were not present on the initial MR-DWI. Diffusion gradients were activated using values of *b* = 0 and *b* = 1000 s/mm^2^. T2-weighted sequences and apparent diffusion coefficient (ADC) maps in the transverse plane were included in the protocol. Comparative studies were included when MR-DWI was performed within 3–5 days after the procedure.

### Study outcomes

The primary outcome involved analyzing of the number and location of new ischemic lesions after carotid stenting, assessing their clinical significance, and observing 30-day major adverse cardiovascular events (stroke, myocardial infarction, death). We investigated the demographic and clinical characteristics of patients undergoing CAS in our center. We assessed the clinical and procedural factors that led to new ischemic lesions.

Secondarily, a comparison of the NIL rates between our center with guided antiplatelet therapy and results from comparative studies was established. The NIL rates in cohorts with standard antiplatelet regimen (acetylsalicylic acid and clopidogrel) were published by Arli et al. [10], Beyhan et al. [11], Bijuklic et al. [12], Pelz et al. [13], Gargiulo et al. [14], and Gensicke et al. [15]. The incidence of NIL in cohorts using platelet function test guided antiplatelet therapy was published by Köklü et al. [9] and Brousallis et al. [7]. In the study by VanHeesewijk et al., the authors did not use platelet function tests, but did use the more potent antiplatelet drug ticlopidine along with clopidogrel [8]. Type of instrumentation used and symptomatology of the stenosis were also reviewed to avoid any potential bias.

### Statistical analysis

Demographic variables were summarized with the use of descriptive statistics. Categorical variables were summarized as counts and percentage, and differences were evaluated with the *χ*^2^ test. Normality of data was tested by the Kolmogorov-Smirnov normality test. The Mann-Whitney *U* test, Kruskal-Wallis test and difference of means test were used for further statistical analyses. *P*-values less than 0.05 were considered statistically significant. SW STATISTICA-TIBCO13.3 was used for analytical processing. SW R (version 4.1.2) was used for the meta-analytical comparison with the historical studies (meta package).

## Results

### Patient and procedure characteristics

**Fig. 1:**
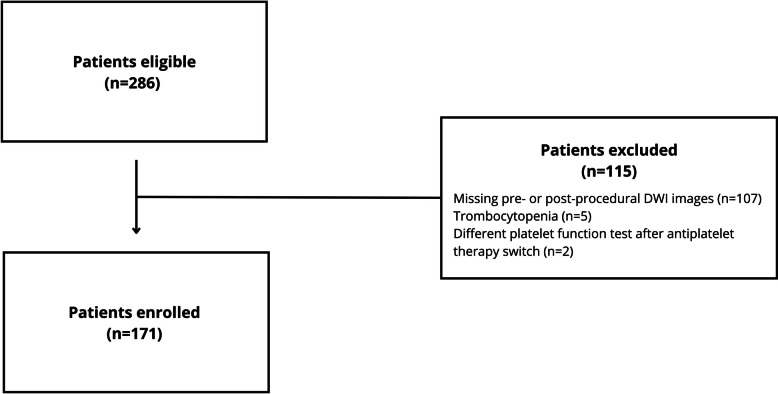
Patient flow diagram

Over a 10-year period, we treated a total of 248 carotid arteries. Exclusion criteria were missing diffusion-weighted imaging before or after carotid stenting, thrombocytopenia and use of different platelet function tests after changing the antiplatelet therapy. Patient flow diagram is shown in Fig. 1.

Finally, 171 patients (median age 71.2 ± 9.2) were enrolled and analyzed. Symptomatic stenosis was treated in 88 patients (51.4%), while asymptomatic stenosis was treated in 83 patients (48.5%). Primary atherosclerotic stenosis was treated in 146 patients (85.4%). Carotid restenosis after previous CEA was treated 25 times (14.6%), occurring between 1 and 30 years after surgery (11 patients were within 2 years of CEA). Four patients who were stented for restenosis after CEA had NIL. In five patients, CT scans were not available for the retrospective evaluation of stenosis length. The degree of stenosis was not assessed in seven patients due to the absence of NASCET evaluation in the report, and CT scans were not available for retrospective analysis. We did not observe any NILs in these patients. Table 1 provides a summary of the baseline clinical and carotid stenosis characteristics in groups of patients with and without NIL. There was no association of the incidence of new ischemic lesions with any demographic characteristics. The incidence of NILs was shown to be associated with the length of stenosis (*p* = 0.024). 74.1% of patients with NILs had a stenosis length 15 mm or more, in contrast to 48.6% of patients without NILs.

**Table 1:** Baseline clinical and carotid stenosis characteristics

**Variable**	NIL present (*n* = 27)	NIL not present (*n* = 144)	*P*
Age, years ± SD	72.7 ± 8.0	71.0 ± 8.3	0.305
Body mass index (kg/m^2^) ± SD	29.1 ± 4.5	28.4 ± 5.2	0.476
Men	20	101	0.680
Stroke history	16	81	0.772
**Comorbidites**			
Hypertension	20	107	0.934
Hyperlipidemia	17	95	0.763
Diabetes	8	50	0.608
Atrial fibrilation	1	13	0.350
Coronary artery disease	7	36	0.950
Chronic kidney disease	4	10	0.175
Smoker	10	75	0.151
Alcohol user	2	9	0.836
**Medication**			
Statins	21	104	0.550
Fibrates	0	3	-
Anticoagulants	3	15	0.914
**Lesions characteristics**			
Symptomatic	17	71	0.193
Grade of stenosis (%) ± SD	80.1 ± 7.4	77.1 ± 9.2	0.104
Length of stenosis ≥ 15 mm	20	70	0.024

Legend: (*n* (%)), *NILs* New ischemic lesions, *SD* Standard deviation, *mm* millimeters

Laboratory-effective DAPT was achieved with clopidogrel in 121 patients (70.7%), ticagrelor in 27 patients (15.7%), ticlopidine in 18 patients (10.5%), and prasugrel in 5 patients (2.9%). This shows that 30% of patients were resistant to clopidogrel.

Closed-cell Wallstent stents (Boston Scientific, USA) were predominantly used in 82 patients (51.4%), Adapt stents (Boston Scientific, USA) in 11 patients (6.4%). Cristallo (Invatec, Italy) and Xact (Abbott Vascular, USA) stents were each used twice (2.4% in total). Palmaz Blue and Palmaz Genesis (Cordis Corporation, USA) stents were each used once. New DWI lesions occurred in 15 patients (15%) with closed-cell stents. Of the open-cell stents, Precise (Cordis Corporation, USA) was used most frequently in 43 patients (25.1%), followed by Acculink (Abbott Vascular, USA) six times (3.5%) and OptiMed Sinus Carotid (Optimed, Germany) three times (1.8%). Ten patients (19.6%) with open-cell stents developed NIL. The double-layered CGuard stent (InspireMD Inc., Tel Aviv, Israel) was used in 20 (11.7%) patients, and NILs occurred in two (10%). NILs occurred in 15% of patients with closed stents and 19.6% with open stents, with no statistical significance in relation to stent type (*p* = 0.607). Other factors such as the use of the protective device, postdilatation and predilatation also showed no correlation with NILs (Table 2).

**Table 2:** Carotid stenting procedure characteristics

		**NIL present (*n* = 27)**	**NIL not present (*n* = 144)**	***P***
**Stent**				
	Closed-cell	15 (15.1%)	84 (84.9%)	0.370
	Open-cell	10 (19.2%)	42 (80.8%)	0.556
	Double-layered	2 (10.0%)	18 (90%)	0.125
**Distal protection**	FilterWire EZ	25 (15.5%)	136 (84.5%)	0.706
**Predilatation**		20 (15.8%)	106 (84.2%)	0.960
**Postdilatation**		16 (14.2%)	96 (85.8%)	0.457
**Length of the procedure (min.) ± SD**		34.8 ± 6.6 (*n* = 16)	34.7 ± 10.0 (*n* = 102)	0.688

Legend: (*n* (%)), *N* total number, *NILs* new ischemic lesions, *DWI* Diffusion-weighted image, *SD* Standard deviation, *min*. minutes

### Analysis of new ischemic lesions

**Table 3:** Localization of new ischemic lesions by cerebral artery basin based on diffusion-weighted imaging

	**Asymptomatic stenoses (*n* = 28)**	**Symptomatic stenoses (*n* = 38)**
**Localisation**		
Frontal lobe	12 (18.8%)	16 (24.2%)
Parietal lobe	7 (10.6%)	11 (16.7%)
Temporal lobe	6 (9.1%)	5 (7.6%)
Occipital lobe	3 (4.5%)	2 (3.0%)
Insular cortex	0	1 (1.5%)
Basal ganglia	0	2 (3.0%)
Cerebellum	0	1 (1.5%)
**Cerebral basin**		
MCA	25 (37.9%)	35 (53%)
PCA	3 (4.5%)	1 (1.5%)
MCA-PCA “watershed”	0	1 (1.5%)
PICA	0	1 (1.5%)

Legend: (*n* (%)), *MCA* middle cerebral artery, *PCA* Posterior cerebral artery, *PICA* Posterior inferior cerebellar artery

**Table 4:** Analysis of new symptomatic DWI lesions after CAS

Case No	Time of symptoms onset	Neurological symptoms	After 1 month
8	0 h (periprocedural)	Mild left-sided hemiparesis	Full recovery, no sequelae
40	0 h (periprocedural)	Moderate paresis of upper limb	Full recovery, no sequelae
49	0 h (periprocedural)	Mild paresis of upper limb	Full recovery, no sequelae
135	1 h	Dysarthria, central paresis of cranial nerve n. VII, plegia of lower limb, paresis of upper limb, neglect syndrome	Slight residual weakness of lower limb
147	0 h (periprocedural)	Plegia of lower limb, paresis of lower limb	Left-sided hemiparesis

Post-procedurally, 66 new ischemic lesions (NIL) were observed in 27 patients (15.8%). NIL occurred in 17 of 88 symptomatic patients (19.3%) and in 10 of 83 asymptomatic patients (12.0%). Twelve patients had only one NIL, five patients had two NIL, two patients had four NIL, three patients had six NIL, and one patient had the highest number of 8 NIL. Fifty-eight (87.2%) NILs were ipsilateral, and eight were contralateral (12.2%). NIL were most commonly located in the frontal lobe (43%) and parietal lobe (27.3%) and when divided according to blood supply, in the middle cerebral artery basin (91%) (Table 3).

New DWI lesions were asymptomatic, i.e., silent ischemia, in 22 (81.5%) patients. Within the first hour after the procedure, we recorded five ischemic cerebrovascular events (2.9%). In three patients, the symptoms of minor stroke disappeared within a few hours, while in two patients with major stroke, decreased strength in the affected limb, or hemiparesis, persisted even after a month (Table 4). In all three patients with short-term symptoms, one NIL was always located cortically, specifically in the postcentral gyrus, cerebellum, and occipital lobe. Patients with persistent neurological deficits had multiple ipsilateral NILs; in one patient, they were located in the postcentral gyrus, and in another, in the frontal lobe and basal ganglia.

In the 30-day periprocedural period following the procedure only two more major cerebrovascular events occurred. On the second day after the CAS, one fatal ipsilateral intracerebral hematoma (ICH) was observed in the patient receiving clopidogrel therapy. This patient was without post-procedural NIL. In the second patient, fifteen days after CAS, a contralateral major stroke occurred. This patient was also receiving clopidogrel therapy. No myocardial infarction was observed. Total incidence of major adverse cardiovascular events in the 30-day periprocedural period was 4.1% (*n* = 7/171). Procedure-related death or stroke rate in our study was 2.9% (*n* = 5/171)

### Comparison of the new ischemic lesions

In the systematic literature review, we identified 10 studies reporting the incidence of NIL after carotid artery stenting using diffusion-weighted imaging, published between 2002 and 2023. These studies also had traceable data about antiplatelet therapy regimens, platelet function test specifics and procedure characteristics. The monitored characteristics are listed in Table 5 [12, 19–27]. There were no study duplicates. The total number of patients in these studies was 3394.

**Table 5:** Procedure characteristic of the comparison studies

Study	Year	Type of study	Number of patients	Total NIL/symptomatic NIL	Predilatation	Postdilatation	Type of stent	Protection device	P2Y12 inhibitor	Platelet function testing
Xu X. et al	**2020**	Retrospective multicenter	694	356/UD	Yes	Yes	Mix	Yes	CLOP	No
Gensicke H. et al	**2015**	Prospective multicentric	124	62/UD	Yes	Yes	Mix	Yes	CLOP	No
Gargiulo G. et al	**2015**	Meta-analysis (*n* = 20)	989	399/UD	Yes	Yes	Mix	Yes	UD	No
Pelz D.M. et al	**2022**	Prospective monocentric	79	36/2	mix	Mix	Open-cell	No	CLOP	No
Bijuklic K. et al	**2013**	Prospective monocentric	728	241/14	UD	UD	UD	UD	CLOP	No
Beyhan M. et al	**2020**	Prospective monocentric	64	20/UD	Yes	Yes	Mix	Yes	UD	No
Arli B. et al	**2023**	Prospective monocentric	99	28/14	Yes	Yes	Mix	Yes	CLOP	No
Köklü E. et al	**2022**	Retrospective monocentric	507	72/UD	Yes	Yes	Mix	Yes	CLOP, TICA	Yes
Van Heesewijk et al	**2002**	Prospective monocentric	72	11/UD	Yes	Yes	Closed-cell	No	CLOP, TICLID	No
Broussalis E. et al	**2019**	Retrospective monocentric	110	8/0	Yes	Yes	Double-layered	Ano	CLOP, TICA, PRA	Yes

Legend: *NILs* New ischemic lesions, *CLOP* Clopidogrel, *TICA* Ticagrelor, *TICLID* Ticlopidine, *PRA* Prasugrel, *UD* Untraceable data

**Fig. 2:**
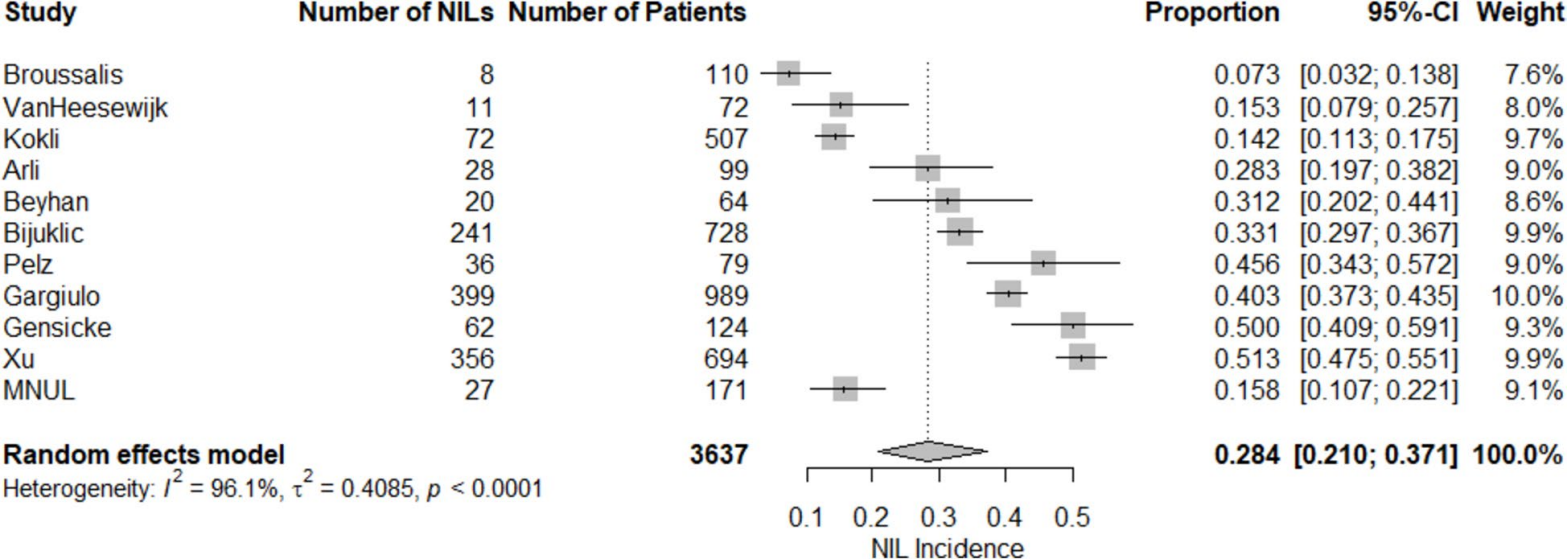
The statistical comparation of the incidence of the new ischemic lesions in comparative studies

The incidence of NIL in our center (15.8%) with guided antiplatelet therapy was compared with comparative studies (Fig. 2, our center as MNUL). The pooled NIL incidence is 28.4% (95% CI 0.210–0.371). Substantial heterogeneity was observed (*I*^2^ = 96.1%), indicating considerable between-institution variability. The incidence observed in our center 15.8% (95% CI 0.107–0.221) was numerically lower than the pooled incidence; however, the confidence intervals overlapped, and therefore no statistically significant difference can be concluded. The incidence in our center was distinctly lower compared to studies with standard antiplatelet therapy: Arli reported 28.3% (95% CI 0.197–0.382), Beyhan 31.2% (95% CI 0.202–0.441), Bijuklic 33.1% (95% CI 0.297–0.367), Pelz 45.6% (95% CI 0.343–0.572), Gargiulo 40.3% (95% CI 0.373–0.435), Gensicke 50.0% (95% CI 0.409–0.591), and Xu 51.3% (95% CI 0.475–0.551).

The incidence of NILs in our center was similar to that in studies using guided or more potent antiplatelet therapy: Köklu 14.2% (95% CI 0.113–0.175) and VanHeesewijk 15.3% (95% CI 0.079–0.257). The incidence of NILs in the study by Broussalis was the lowest, at 7.3% (95% CI 0.032–0.138).

## Discussion

In this study, the incidence of new ischemic lesions after carotid stenting was 15.8%. Most of the new ischemic lesions (81.5%) were asymptomatic. The procedure-related stroke/MI/death incidence was 2.9% in a 30-day periprocedural period proving the safety of guided antiplatelet therapy in carotid stenting.

The only risk factor associated with new ischemic lesions in our cohort was the length of the stenosis. Previously published studies and meta-analyses have shown that older age, diabetes mellitus, significantly calcified or ulcerated plaque, length of stenosis, and predilatation are independent risk factors for the development of silent ischemic lesions during CAS [10–13]. The best thresholds for predicting new ischemic lesions were identified as age ≥ 68 years and stenosis length ≥ 15 mm in one study [28]. In this study, we confirmed a statistically higher rate of NIL in stenoses with a length ≥ 15 mm. This may be a factor to consider preoperatively when selecting a stent design. The use of closed-cell stents (compared to the use of open-cell stents) significantly reduces the incidence of new DWI lesions, while the use of open-cell stents predisposes to a 25% higher chance of developing postprocedural new ischemic lesions on MR-DWI [16, 21, 24, 29, 30]. In this study the incidence of NIL was higher in patients with open-cell stents implanted (19.6%) than in the patients with closed-cell (15%) and double-layer stents (10%). However, this trend was not statistically significant (*p* = 0.574). It has been shown that the use of distal protection devices significantly reduces the incidence of new DWI lesions [31, 32]. In our cohort, we were unable to place protective devices in 10 patients (5.8%), and we recorded two NIL in those patients.

Most NIL in this study were located ipsilaterally and only 12.2% were located contralaterally. We consider atherosclerotic involvement of the aortic arch and instrumentation during the procedure to be the main causes of contralateral NIL. The development of new ischemic lesions is determined not only by the characteristics of the lesion itself, but also by the experience of the surgeon and the use of selected instruments.

We also demonstrated that clopidogrel resistance is common in patients undergoing carotid stenting as 30% of patients were resistant to clopidogrel and had been switched to ticagrelor or ticlopidine. In our opinion, medical therapy during the procedure is as important as the correct patient selection and safe performance of the procedure. Recent publications confirm clopidogrel resistance as a potential predictive factor for thromboembolic complications in CAS and state ticagrelor as a suitable alternative [13–17].

In the identified comparative studies, the incidence of NIL on DWI after CAS ranged from 7.3% to 51.3% [12, 19–27]. The incidence of NIL in this study was 15.8%. Only two comparative studies had an incidence of NIL below 16%, these studies were characterised by guided antiplatelet therapy [19, 21]. Köklü et al. in a 2022 study in a large sample of patients demonstrated the incidence of NIL was 14.2%. The authors used predilatation, postdilatation, and embolic device protection during the procedure as in other historical studies. The only difference in the approach was in the pre-procedural testing of clopidogrel efficacy and, if resistance was identified, changing to ticagrelor at a saturating dose of 2 × 90 mg and a maintenance dose of 90 mg [21]. A 2019 study by Brousallis et al. in a sample of 110 symptomatic and asymptomatic patients investigated the efficacy of the new Casper double-layer stent (MicroVention, Inc., California, USA). In addition to the use of the double-layer stent, which was developed to reduce the risk of embolic complications, the clopidogrel dose was increased to 150 mg daily in clopidogrel-resistant patients. Alternatively, prasugrel 10 mg daily or ticagrelor 90 mg twice daily was administered. In this study, only 7.3% of patients (8/110) had NIL post-procedurally [19]. These findings suggest that using a double-layered stent alongside guided antiplatelet therapy results in the lowest incidence of new ischemic lesions and periprocedural major adverse cardiovascular events [10, 28].

Van Heesewijk et al. reported an incidence of NIL of 15.3% in a small cohort of patients (*n* = 72) receiving clopidogrel or ticlopidine without platelet function testing [20]. The proportion of patients receiving each drug is not traceable from the published data; therefore, a direct causal attribution of the low NIL rate to the use of ticlopidine cannot be established. Other factors may have contributed to this favorable outcome, including the relatively small cohort size or the closed-cell type of stent used. We decided to keep this study in the comparison, as it does not contradict the findings of our work. In the comparing historical studies with standard antiplatelet therapy regimen, the incidence of NIL ranges from 28.3 to 51.3% [22–27]. The difference in the incidence of NIL between guided antiplatelet therapy and standard antiplatelet therapy was statistically significant.

The first limitation of our study is its retrospective and single-center nature, along with a relatively small patient population. Some factors (open-cell stent, stenosis symptomatology) might have been significant in the development of NIL if there were a larger cohort. The incidence of NIL was higher in a group of patients with open-cell stents than in those with closed-cell and double-layer stents; however, this trend was not significant. We used various types of platelet function tests from different manufacturers, which may have introduced variability.

## Conclusion

Guided antiplatelet therapy in carotid stenting is safe and is associated with a low incidence of major adverse cardiovascular events. The incidence of the new ischemic lesions in cohorts with guided antiplatelet therapy is significantly lower compared to the results with the standard antiplatelet therapy regimen. In our small cohort with guided antiplatelet therapy, new ischemic lesions after carotid stenting were associated with a length of the stenosis ≥ 15 mm. Further prospective and multicenter studies are warranted to validate our results, define standardized platelet function testing protocols, and assess the long-term cognitive and neurological outcomes associated with guided antiplatelet therapy in carotid stenting.

## Data Availability

The datasets used and/or analyzed during the current study are available from the corresponding author on reasonable request.

## Abbreviations

LTA: Light transmission aggregometry
PRA: Prasugrel
TICLID: Ticlopidine
TICA: Ticagrelor
CLOP: Clopidogrel
NASCET: North American Symptomatic Carotid Endarterectomy Trial
MI: Myocardial infarction
DAPT: Dual antiplatelet therapy
TIA: Transitory ischemic attack
MRI: Magnetic resonance image
DWI: Diffusion-weighted image
NIL: New ischemic lesion
CEA: Carotid endarterectomy
CAS: Carotid stenting
